# Comparative Evaluation of the Adhesive Properties of Two Generations of Dentin Bonding Agents by Checking the Microleakage in the Primary Teeth: An *in vitro* Study

**DOI:** 10.5005/jp-journals-10005-1109

**Published:** 2011-04-15

**Authors:** Sajjad Hasim Mithiborwala, Vishwas Chaugule, Autar Munshi, Vishwas Patil

**Affiliations:** 1 Associate Professor, Department of Pedodontics and Preventive Dentistry, Terna Dental College and Hospital, Mumbai, Maharashtra, India; 2 Head and PG Guide, Department of Pedodontics, DY Patil Dental College and Hospital, Pune, Maharashtra, India; 3 Dean, Head and PG Guide, Department of Pedodontics, KD Dental College and Hospital, Mathura, Uttar Pradesh, India; 4 Associate Professor, Department of Pedodontics, DY Patil Dental College and Hospital, Pune, Maharashtra, India

**Keywords:** Dye penetration, Interexaminer variability, Interfacial morphology, Microleakage, Total-etch system, Self-etch system.

## Abstract

Early childhood caries is now affecting the children in dangerous proportions. There is a widespread loss of the tooth material irrespective of the type of the carious lesion. Restoration of such lesions with a strong permanent bond between the dental tissues and the restorative dental materials would be a highly desirable requisite of any restorative material. Ultramorphological characterizations show that the interfa-cial morphology and the chemical characterization of the bonding systems appear to be strongly associated with each other and, therefore, observing and understanding the interfacial phenomenon and its quality would be of great importance in the selection of a dental adhesive for its use in pediatric restorative dentistry.

## INTRODUCTION

Early childhood is marked by tremendous growth and development of the face and the dentition, both of which require regular monitoring and supervision of a dental professional. Among the more common oral conditions, early childhood, dental caries is the preeminent concern because of its tremendous prevalence and consequences. Overall, nearly one in five (18.7 %) US children between the ages 2 to 4 have experienced visually evident tooth decay.^1^

National surveys conducted during the past three decades have demonstrated a decline in the overall mean levels of clinically detectable dental caries in the US children and adolescents.^2^ Nevertheless, dental caries remain the single most common disease of childhood that is neither self-limiting nor amenable to short-term pharmacological management. More than 80% of the pediatric population is affected by dental caries by age 17. In a study conducted by Balwant Rai et al (2007) the mean DMFT was found to be 2.82, 2.87, 3.40 and 3.15 in 9, 10, 11 and 12-year-old children, while the mean DMFS was found to be 3.82, 3.87, 3.76 and 4.26 respectively.^3^

The operative options are significant as the action is irreversible and restorations have a finite life span. Such a decision assumes that an active caries lesion is present and that no other more conservative therapy is possible to affect a successful outcome. Traditional cavity preparation includes varying degrees of ‘extension for prevention’ in an attempt to remove adjacent caries-prone tooth structure. Once the lost tooth structure has been replaced and the tooth has been restored to its original shape with a restorative material, it is assumed that the tooth functions as a trouble free component in the oral cavity. But the irony remains that it does not usually happen, as there is no dental restorative material that matches the qualities of dental enamel. In addition to failing to match the properties of dental enamel and dentine, the problems due to nonadhe-siveness of such restorative materials to the tooth structure remain a cause of further concern to the dentist.

Barnes JC and Henson JL^4^ (1984) suggested that clinicians and researchers used microleakage as a measure for assessing the performance of restorative materials in the oral environment. The importance placed on this measure was based on the premise that no available restorative material was perfectly adaptive or adhesive to the tooth. Numerous investigations have used a variety of research tools to evaluate the extent of microleakage and the marginal integrity of the restorations. Dye penetration measured on the sections of restored teeth is the most common technique for evaluating microleakage at the tooth restoration interface. Many controversies have surfaced in the literature when comparing *in vivo* and *in vitro* microleakage testing and whether the results from *in vitro* investigations can be applied to clinical situations. Barnes DM et al^5^ (1993) reported that *in vitro* studies are more prone to dye penetration at the resin composite tooth interface than *in vivo* studies.

Longevity of restorations is very low in the primary dentition.^6^ Generally, the earlier the age at restoration, the lower the longevity.^7,8^ The predicted life span of restorations is even shorter.^7^ The vast majority of clinical research on the primary dentition from the systematic review involves relatively short-term comparisons of dental materials, particularly newer proprietary materials, as they enter the marketplace. Qvist et al found that the major reasons for replacement of restorations in the primary dentition were restoration fracture or total loss.^9^ There is a continuing search, to the present day, for improved materials as a restorative solution to caries management in the primary dentition.

Marginal leakage cannot be eliminated even when higher shear bond strength is obtained for some adhesive systems. Thus, it can be assumed that the magnitude of bond strengths is not the only predictor of the sealing ability. So the development of bonding systems, which will provide a true and stable adhesive bond to tooth structure in the rigors of the oral environment, is a high priority.

The widespread demand for and the use of dental adhesives have thus fueled an intense development of better and easier dental adhesives in rapid succession. The ‘generational’ definitions help in the identification of chemistries involved, the strength of dentinal bond and the ease of use for the practitioner. The fundamental principle of adhesion or bonding to the tooth substrate is an exchange process where inorganic tooth material is exchanged for a synthetic resin. Though bonding to the enamel could be effectively achieved, bonding to dentin was a real challenge due to its heterogeneous nature and presence of water, presence of the smear layer and smear plugs, etc. All these created a significant problem, because they prevented direct contact of dentin bonding agent to the dentin surface.

In a very short period of time single-bottle agents, acid primers and self-etching bonding systems became popular. However, these products should be evaluated and tested before they are used on a large-scale basis. Bonding systems have gone through a number of generational advancements and overcome the early hiccups. Today, we have the bonding systems capable of providing predictable and durable bonding to both enamel and dentin with easy to use approaches.

Until recently, all adhesive systems used in the past had three steps prior to restoration. These involved etching, priming and bonding. This was quite cumbersome. Hence, the thought process continued in the direction of reducing the number of steps involved in bonding prior to the restoration with better clinical results. Currently, there are two philosophies on simplification of the adhesive systems, viz:

- The total-etch systems, with a separate etchant and a primer/adhesive and
- The self-etching systems, which combines etching and priming in one bottle and have a separate adhesive agent or which combine all three steps in a single solution.

Studies comparing the total-etch systems and self-etching systems showed results varying from no significant difference to higher or lower bond strength and sealing ability in primary dentition than in permanent dentition.^10,11^ Results of recent *in vitro* studies have revealed the lower efficacy of self-etch system than the total-etch system in primary dentition. Chemical, physiological and micro-morphological differences such as decreased mineralization, small size and lower concentration of dentinal tubules, decreased permeability and more reactivity of primary dentin to acidic conditioner were thought to be responsible for lower bond strength and sealing ability in primary dentition.^12^ Despite simplification of bonding systems, technique sensitivity and substrate variability; concerns about enamel and dentin bonds have increased.

In the light of these developments, this study was undertaken to compare the behavior of both; the 5th generation bonding system (Prime and Bond NT) and the 6th generation bonding system (Xeno III) by examining their microleakage in primary teeth thereby determining the integrity and quality of bonding at the interface.

## MATERIALS USED IN THE STUDY

- Bonding agents: Prime and Bond NT (LOT - 051123 2007-11) and Xeno III (LOT - 0605000856-857 200804) bonding adhesives
- HILUX dental curing light - Kulzer, Benlioglu Dental Inc. Turkey. Order no - 950-200-230, Class II equipment
- Gold Palladium Sputtering Unit: JEOL JFC 1600 auto fine coater. Tokyo, Japan
- Analytical scanning electron microscope―JSM 6360 -A, JSM 6360 - LA
- Double-sided diamond disks procured from Dental World, Pune
- 0.5 % chloramine T powder
- Thermocycling units (Hot water bath - maximum temperature of 100 °C and Cold water bath - minimum temperature of 4 °C) manufactured by Vilman Industries, Pune
- Sony digital SLR (A 100 W) camera
- Small straight fissure diamond abrasive point (Ex 41 -Mani).

## METHODOLOGY

A total of 24 human primary molars, which were indicated for extraction, were collected for the study purpose. They were stored in 0.5% chloramine T solution at 37°C until further procedures.^13^

The teeth were then equally distributed into two groups each namely C1―Prime and Bond NT and C2―Xeno III.

### Selection Criteria for Cavity Preparation

Selection of the area for cavity preparation was done in such a way that the cavity would house in sound dentin structure.

### Selection of the Bur

Small straight fissure diamond abrasive point (Ex 41 -Mani) with a height and diameter measuring 2 and 1 mm respectively was selected because primary enamel is usually 1 mm in thickness and the primary dentin is 2 to 3 mm in thickness.^14^

### Procedure^15^

**Fig. 1:**
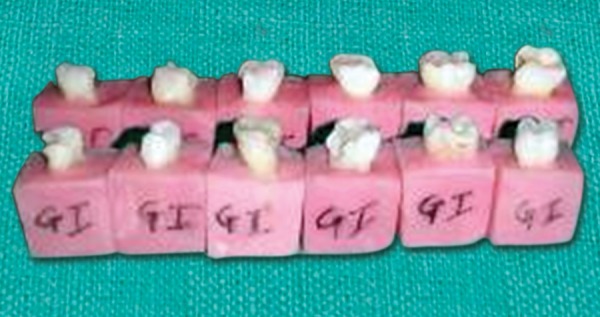
Cavity preparation followed by bonding and composite restoration in group C1

**Fig. 2:**
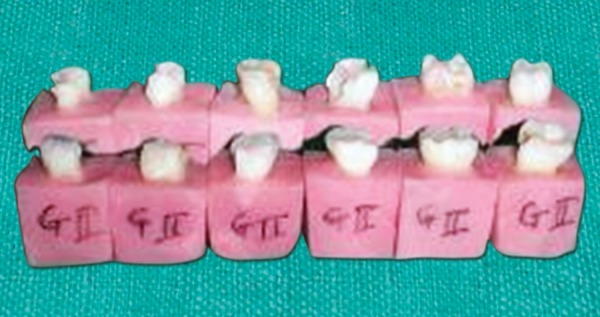
Cavity preparation followed by bonding and composite restoration in group C2

**Fig. 3:**
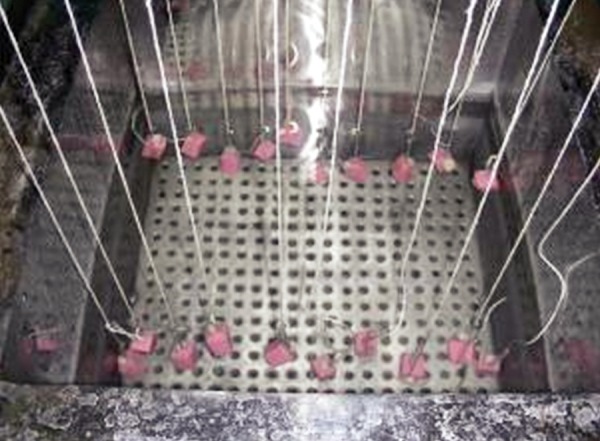
Thermocycling of the specimens

**Fig. 4:**
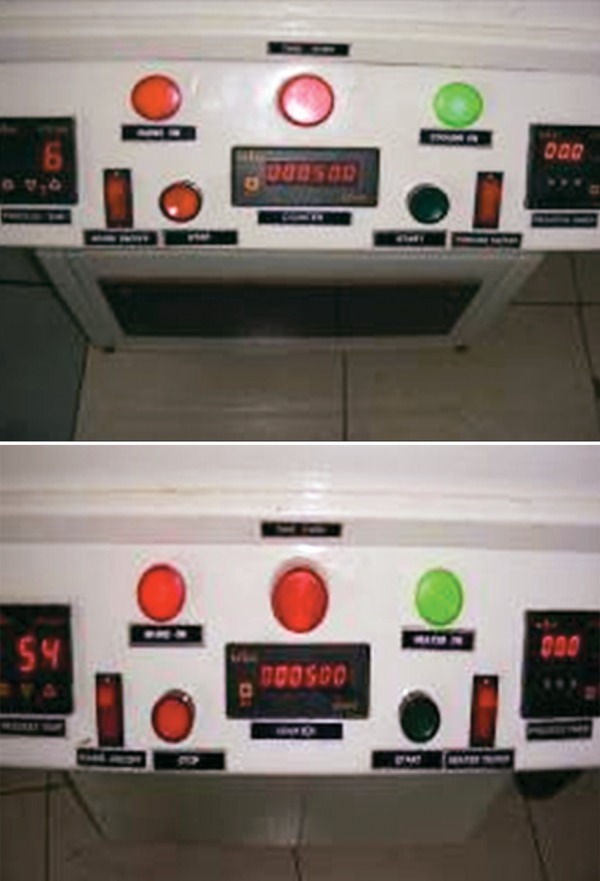
Readings of the temperature and number of the cycles completed in the thermocycling units

**Fig. 5:**
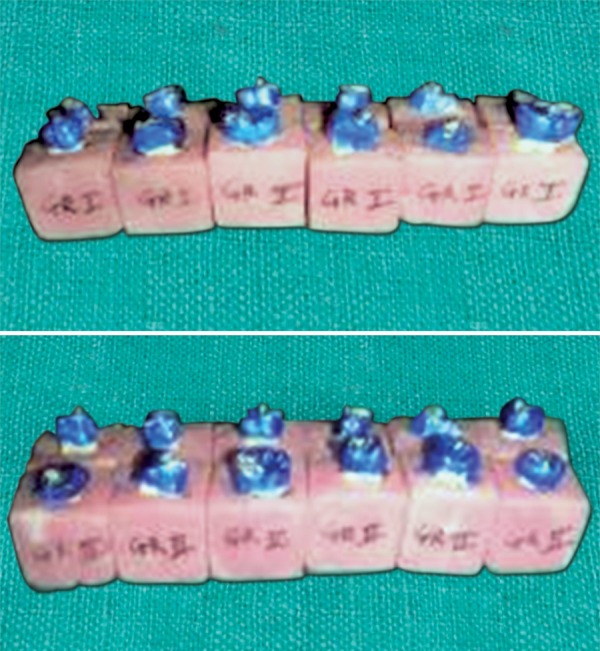
Thermocycled specimens with nail polish coating

**Fig. 6:**
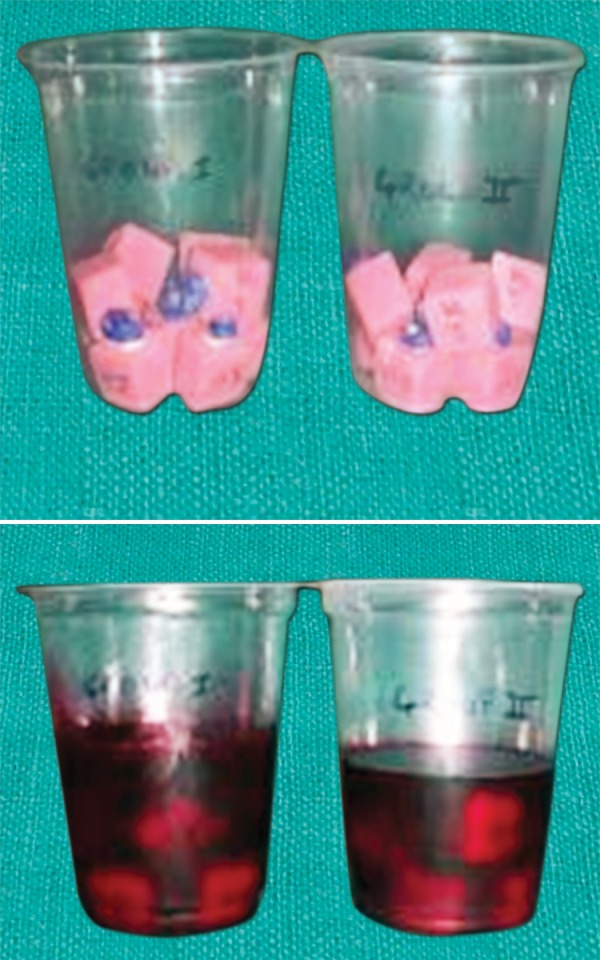
Immersing the specimens in 0.2% basic fuchsine dye

**Fig. 7:**
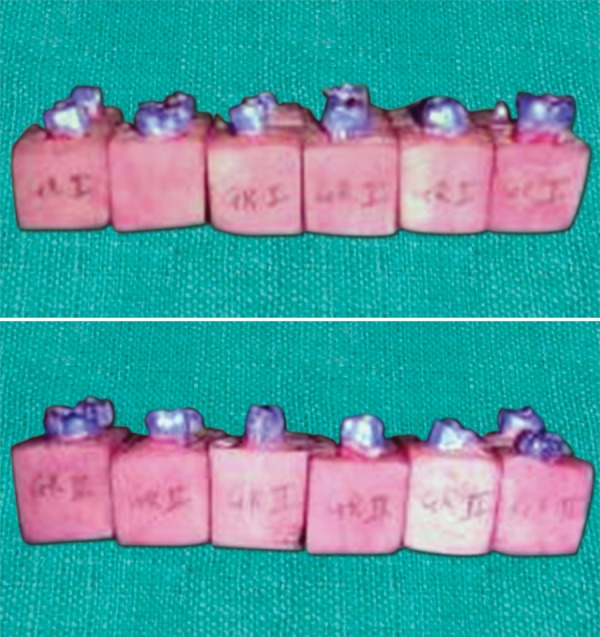
Following immersion application of sticky wax and clear nail polish

**Fig. 8:**
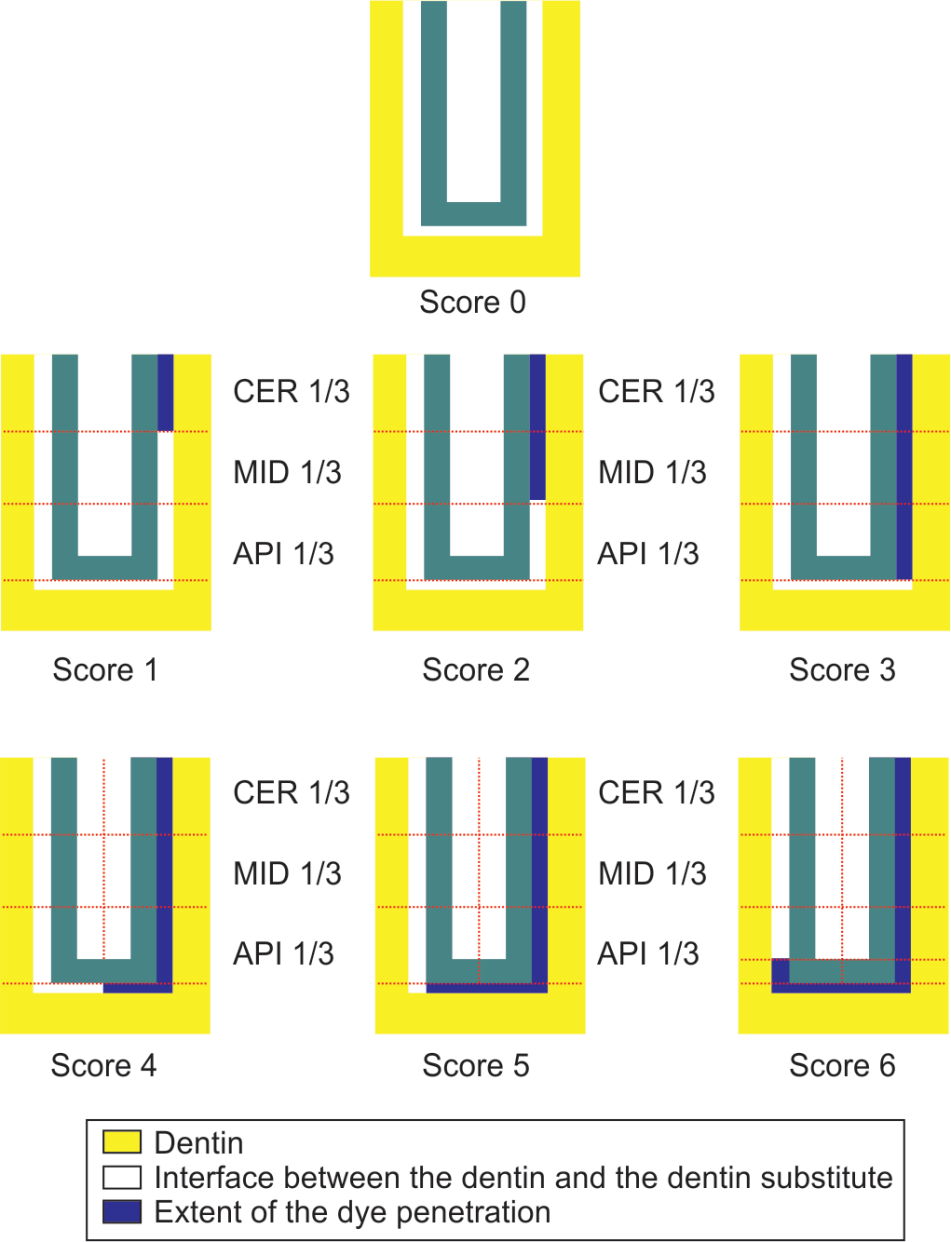
Dye penetration scoring criteria

Left over apices of the roots were sealed and the teeth were mounted in acrylic resin blocks prepared using L-Moulds. Occlusal cavities were prepared by using the small straight fissure diamond abrasive point (Ex 41 - Mani). Toilet of the cavity was performed by cleaning with RC Prep for 1 minute, followed by rinsing thoroughly with 3% sodium hypochlorite using 5 cc syringe with 24-gauge needle.^16,17^ The prepared specimens were then rinsed thoroughly with distilled water. The cavities were air dried.^18^ The bonding procedure for both the groups was carried out according to manufacturer’s instructions. Cavities were restored with a composite (Figs 1 and 2). The specimens were kept in water at room temperature for 24 hours. The teeth were then thermocycled for 500 cycles with a dwell time of 30 seconds at 55°C and 5°C each (Figs 3 and 4). The teeth were then coated with colored nail polish 1mm short of the restoration margins (Fig. 5) and then immersed for 24 hours in 0.2 % basic fuchsine dye (Fig. 6). Following removal from the dye solution, the specimens were rinsed in tap water. Prior to sectioning, the entire area was protected by sticky wax application followed by clear nail polish (Fig. 7). Sectioning was done in a buccolingual direction through the center of the restoration using a double-sided diamond disk with a continuous spray of water.^15^ The sections thus obtained were polished using silicon carbide paper of size 320 and 600 grit. Diamond paste was used for final finishing.^19^ The sections were rinsed thoroughly with 3% sodium hypochlorite and washed gently under the running tap water and were air-dried later. The sections were photographed using digital SLR camera. All the sections were examined independently and critically evaluated by three different examiners. All the samples were scored according to the predetermined scoring criteria^20^ (Fig. 8).

### Score

1. No penetration of the dye      0
2. Penetration of the dye upto the end of the cervical one-third      1
3. Penetration of the dye upto the middle third section      2
4. Penetration of the dye upto the apical third section      3
5. Penetration of the dye crossing the apical third and going upto the basal half interfacial length      4
6. Penetration of the dye beyond the basal half, reaching upto the other apical third region      5
7. Crossing over on the other side of the apical third area      6

## RESULTS

### Sections of the Specimens from the Group C1 to show Microleakage

**Fig. 9:**
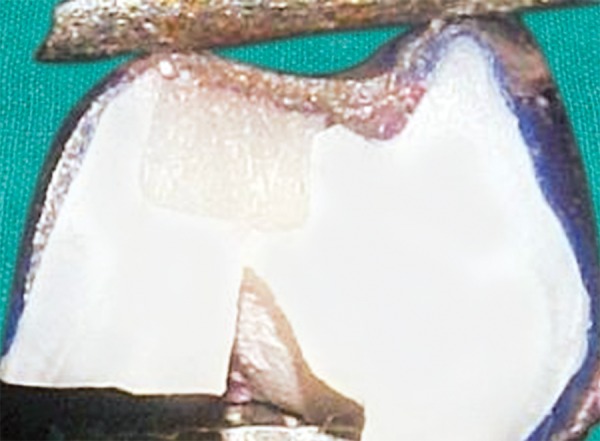
Dye penetration with score 0

Figure 9 shows the buccolingual section of the sample from the group C1 showing no dye penetration with score 0.

**Fig. 10:**
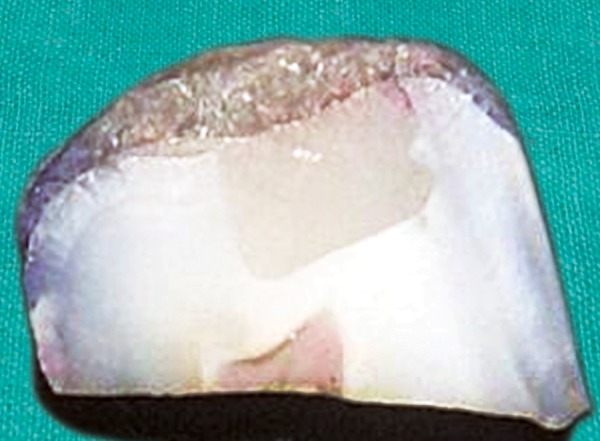
Dye penetration with score 1

Figure 10 shows the sections of the samples from the same group with score 1 wherein the dye penetration is till the level of coronal one-third.

### Sections of the Specimens from the Group C2 to show Microleakage

**Fig. 11:**
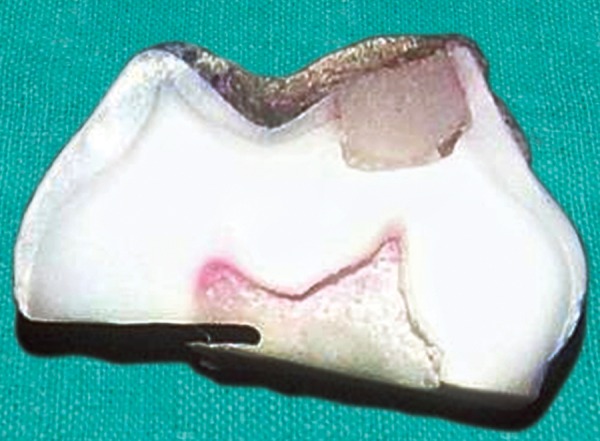
Dye penetration with a score 2

Figure 11 shows the buccolingual section of the sample from the group C2 showing the dye penetration upto the middle third region with score 2.

**Fig. 12:**
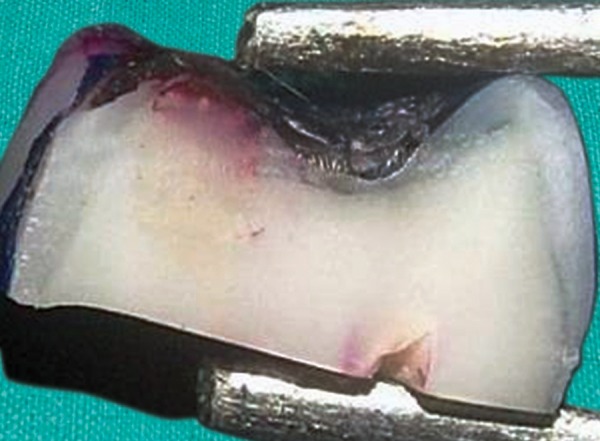
Dye penetration with a score 3

Figure 12 shows a section of the sample from the group C2 with score 3 where the penetration of the dye is seen upto the apical third section of the restored cavity.

## STATISTICAL ANALYSIS

The data, thus, collected was entered into MS-Excel worksheet for the statistical analysis as per the ’Statistical Package for Social Sciences’ (SPSS) software. The results were represented in the form of tables and graphs.

To determine the quality of bonding by checking the microleakage at the interface―Kendall’s coefficient of concordance (W) was applied to check the interexaminer variability for microleakage scores and Mann-Whitney test was later applied to determine the depth of microleakage.

p-value was determined at 95% confidence limits for all the tests mentioned above.

Tables 1 and 2 show the rating scores of the dye penetration recorded by the three different examiners for the 12 samples in each group.

Tables 3 and 4 show the interexaminer variability between the groups C1 (Prime and Bond NT) and C2 (Xeno III) that was determined statistically from the value of Kendall’s coefficient of concordance ‘W’. The interex-aminer variability in terms of scoring of the results was not found to be statistically significant in both the groups with p-values as follows:

Group C1 with p-value = 0.878 (p > 0.05)

Group C2 with p-value = 0.135 (p > 0.05)

**Table 1:** **Table 1:** Microleakage scores in group C1 (Prime and Bond NT)

*Sample no*		*Score*					
		*Examiner 1*		*Examiner 2*		*Examiner 3*	
1		0		0		0	
2		1		1		1	
3		0		0		1	
4		1		1		0	
5		0		1		0	
6		3		3		4	
7		4		3		5	
8		6		6		6	
9		1		1		1	
10		3		3		2	
11		1		2		0	
12		5		5		5	

**Table 2:** **Table 2:** Microleakage scores in group C2 (Xeno III)

*Sample no*		*Score*					
		*Examiner 1*		*Examiner 2*		*Examiner 3*	
1		1		1		0	
2		3		3		3	
3		2		2		2	
4		4		0		4	
5		4		4		4	
6		4		3		4	
7		4		3		4	
8		2		2		1	
9		2		2		2	
10		2		2		2	
11		1		0		1	
12		4		4		4	

**Table 3:** **Table 3:** The mean rank calculated for each examiner from the microleakage scores seen in Tables 1 and 2 in both the groups

*Groups*		*Examiners*		*Mean rank*	
Prime and Bond NTC1		Examiner 1		1.96	
		Examiner 2		2.08	
		Examiner 3		1.96	
Xeno IIIC2		Examiner 1		2.25	
		Examiner 2		1.75	
		Examiner 3		2.00	

**Table 4:** **Table 4:** The calculated ‘W’ value for both the groups after applying Kendall’s ‘W’ test statistics from the above calculated mean rank seen in Table 3

*Groups*		*Test statistics*			
Prime and Bond NTC1		Number of samples		12	
		Kendall’s W		0.011	
		p-value		0.878	
Xeno IIIC2		Number of samples		12	
		Kendall’s W		0.167	
		p-value		0.135	

Therefore, the scoring of the Examiner No. 1 was taken up for the purpose of inference in both the groups (Table 5).

**Table 5:** **Table 5:** The microleakage scores as given by the examiner 1 for each group respectively

*Sample no*		*Score (examiner 1)*	
1		0	
2		1	
3		0	
4		1	
5		0	
6		3	
7		4	
8		6	
9		1	
10		3	
11		1	
12		5	
*Sample no*		*Score (examiner 1)*	
1		1	
2		3	
3		2	
4		4	
5		4	
6		4	
7		4	
8		2	
9		2	
10		2	
11		1	
12		4	

## STATISTICAL RESULTS

### Mann-Whitney Test Statistics

From the value of ‘U’, the p-value came to 0.206 (p > 0.05) which means that the microleakage is not statistically significant when compared with the scores, noted in both the groups C1 and C2 as seen in Table 6.

**Table 6:** **Table 6:** Mean rank calculated for each examiner

*Mann-Whitney* *significance* *‘U’ value*		*p-value*		*Level of*	
50.500		0.206*		NS	

NS: not significant; * arithmetic precision used to round significant digits after the decimal point.

**Fig. 13:**
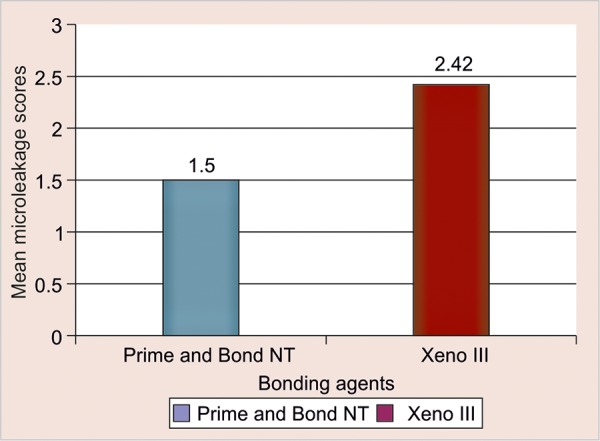
Relationship of the mean scores of dye penetration in both the groups

Figure 13 shows the relationship of the mean scores of dye penetration (microleakage) in both the groups C1 (Prime and Bond NT) and C2 (Xeno III). The group C1 (Prime and Bond NT) had a mean score of 1.5, whereas in the group C2 (Xeno III) the mean score was 2.42.

## RESULTS

From the statistical analysis of the microleakage (dye penetration) study, there was no statistically significant difference between the two groups with p-value of 0.206 (p > 0.05), but from the mean value of both the groups, it may be concluded that the marginal leakage may be more in group C2 (Xeno III) when compared with group C1 (Prime and Bond NT).

## DISCUSSION

There is a plethora of information available on the mechanism of adhesion for bonding systems on permanent teeth. Primary teeth are smaller in size, has thinner enamel - dentin and show a rapid spread of dental caries. Hence, with less tooth structure available for bonding of composite resin material, proper dentin bonding steps should be followed for success of the composite restoration in primary teeth. Achievement of a consistently reliable, gap-free and complete attachment of resin composite to dentin is of profound importance in restorative dentistry. Formation of an acid resistant, resin impregnated hybrid layer seems to depend on the penetrating ability of resin into etched dentin surface and also on conditioning and permeability of dentinal surface.^21,22^ However, the similar type of information on primary teeth appears to be scanty. Few studies in the past stated the presence of formation of thicker hybrid layers^23^ in primary teeth with shorter resin tags.

Hence, the current study was performed *in vitro* to study the sealing ability and microleakages of the total-etch (group C1 - Prime and Bond NT) and self-etch adhesive (group C2 - Xeno III) systems on primary teeth.

In the present study, it may be concluded from the mean of microleakage scores of both the groups that the marginal leakage may be more in group C2 (Xeno III) when compared with group C1 (Prime and Bond NT) but there was no statistically significant difference between the two groups. The results of the present study are complementary to the results reported by Stalin A, Varma BR et al^24^ (2005) who evaluated the tensile bond strength, fracture mode and microleakage of fifth generation (single bond -group 1) and sixth generation bonding system and found no statistically significant difference between two groups thus concluding that, the self-etching adhesive is better for bonding in primary dentition. Van Meerbeek B et al^25^ (2003) also demonstrated the basic bonding mechanism to enamel and dentin by means of ultramorphological and chemical characterization of tooth biomaterial interfacial interactions. They reported that a self-etch approach may have the best future perspective. Clinically, when adhesives no longer require an ‘etch and rinse’ step, the application time, and probably more importantly the technique sensitivity are substantially reduced which is advantageous in the field of pediatric dentistry.

## CONCLUSION

Since, the reduction in the technique sensitivity of any bonding system would always be a preferred factor in pediatric restorative dentistry; further studies should be carried out keeping in mind the above variables toward the development of a universal bonding system. Therefore, after having gone through the review of literature and the study conducted with its limitations, it appears that the inclination toward the selection of the bonding system may lean toward the self-etching bonding system. The candid recommendation for the use of any specific bonding system in pediatric dentistry seemed to be difficult at this juncture due to the availability of the limited resources at the time of conducting the study.
